# More Levels of Complexity in the Control of Intestinal Inflammation

**DOI:** 10.1016/j.jcmgh.2021.06.009

**Published:** 2021-06-25

**Authors:** David H. Adams

**Affiliations:** The National Institute for Health Research Biomedical Research Centre, University Hospitals Birmingham NHS Foundation Trust, Centre for Liver and Gastrointestinal Research, Institute of Immunology and Immunotherapy, University of Birmingham, Birmingham, United Kingdom

The article by Osorio-Barrios et al^1^ describes a new and provocative twist to our understanding of how the homing of lymphocytes to the gut is controlled. The current paradigm suggests that the chemokine receptor C-C chemokine receptor 9 (CCR9) promotes CD4 homing to the small bowel and, although not required for T-cell homing to the normal colon, it does plays an important role in the generation and maintenance of colitis in animal models and human disease. CCR9 is a G-protein–coupled receptor whose only ligand is the chemokine C-C chemokine ligand 25 (CCL25). CCL25 is detected on mucosal blood vessels in the small bowel and in the inflamed colon where it triggers migration of CCR9-expressing cells into gut tissue. These findings have led to the evaluation of CCR9 inhibitors in patients with inflammatory bowel disease.^2^

In the current article, Osorio-Barrios et al^1^ suggest that the lymphocyte receptor providing gut tropism for effector CD4 T cells in colitis is not CCR9 but a complex formed by CCR9 and another G-protein coupled receptor: the dopamine receptor D5 (DRD5). The implication of dopamine is interesting because it is present at high levels in normal gut tissue but decreased in response to inflammation, and immune cells express high- and low-affinity dopamine receptors, allowing them to respond to changes in tissue levels of dopamine.^3^

Osorio-Barrios et al^1^ used standard in vivo models in which colitis was induced in mice either by transfer of naïve T cells into Rag1-deficient mice or by administration of dextran sulfate. They then studied the tissue distribution and inflammatory response after the adoptive transfer of congenic T cells, including those lacking DRD5 or CCR9, allowing them to dissect the role of CCR9 and DRD5 on T-cell homing and differentiation in vivo. They report that colitis is attenuated to a similar degree when T cells lack either CCR9 or DRD5. This is associated with a reduction in CD4 T-cell infiltration in the gut but not in changes in T-cell activation, suggesting that the effect is owing to reduced gut homing. Furthermore, DRD5-deficient T cells show a selective failure to migrate into the gut while migrating normally into other tissues despite expressing high levels of CCR9. This finding led them to explore whether DRD5 and CCR9 might form a heterodimeric receptor and to establish whether this heterodimeric complex is the true gut-homing receptor. They used a combination of molecular imaging techniques to show that DRD5 can form a complex with CCR9, but not another chemokine receptor C-X-C chemokine receptor type 4 (CXCR-4), and then used receptor activation approaches to show that a DRD5 antagonist could inhibit signaling through CCR9. They then determined the transmembrane domains involved in heterodimerization before showing that disruption of the heterodimers abrogated gut homing of CD4 T cells. Finally, and crucially, they show that the heterodimers are present in mouse and human gut tissue.

Why is this study important? It has been known for many years that G-protein coupled receptors including chemokine receptors can form heterodimers but a complex involving a chemokine receptor and a dopamine receptor has not been reported before.^4^ It is important this work is confirmed in other studies given the difficulty in showing the biological significance of chemokine-receptor heterodimers but the authors have gone to some lengths to exclude artefacts and make sure their findings have functional significance. It will be interesting to investigate other heterodimeric complexes, the authors report that CXCR4 does not interact with DRD5 but what about other chemokine receptors for example CCR6 which has a role in recruiting T-helper 17 cells to the gut? The concept that T cells can use the same receptor to sample distinct signals is fascinating and adds another layer of complexity to the regulation of immune responses and inflammation. It emphasizes the importance of multiple environmental cues in shaping local immunity and response to injury. The role of dopamine in this context is particularly interesting given its ability at high tissue concentrations to activate low affinity receptors that promote immunosuppression and help maintain homeostasis whereas a fall in tissue levels, such as is seen with inflammation, selectively activates high-affinity pro-inflammatory receptors such as DRD5.

The authors mention the therapeutic potential that comes from this work and a greater understanding of the biological significance and the complexity of responses driven through such networks will help inform strategies to target these novel pathways.
